# Navigating self-injury in a digital world: adolescents’ perspectives on coping, help-seeking, and technology

**DOI:** 10.3389/fdgth.2026.1905943

**Published:** 2026-08-26

**Authors:** Israel Ramirez, Olga Barnas, David C. Mohr, Madhu Reddy, Jason J. Washburn, Theresa Nguyen, Kaylee P. Kruzan

**Affiliations:** 1 Department of Preventive Medicine, Northwestern University Feinberg School of Medicine, Chicago, IL, United States; 2 Department of Informatics, University of California Irvine Donald Bren School of Information and Computer Sciences, Irvine, CA, United States; 3 Department of Psychiatry and Behavioral Sciences, Northwestern University Feinberg School of Medicine, Chicago, IL, United States; 4 Mental Health America, Alexandria, VA, United States

**Keywords:** adolescents, digital intervention, self-harm, self-injury, technology use

## Abstract

**Background:**

Nonsuicidal self-injury (NSSI), hereafter referred to as self-injury, is common among adolescents and is associated with elevated risk for suicide attempts and other adverse mental health outcomes. Because most adolescents who self-injure do not receive formal treatment, accessible alternatives are needed. Digital mental health interventions (DMHIs) may offer an accessible and scalable means of support. While previous research has examined DMHIs for youth, less is known about how adolescents who engage in self-injury understand their experiences and use digital technologies, including emerging tools such as artificial intelligence (AI), to support their mental health. This study explored adolescents’ lived experiences of self-injury, help-seeking behaviors, and technology use for coping and support.

**Methods:**

We conducted semi-structured interviews between May 2024 and April 2025 with 21 adolescents (14–18 years old) with lived experience of self-injury. Recruitment occurred through a mental health screener hosted on a large national mental health nonprofit's website. Interviews explored adolescent experiences with self-injury, barriers to seeking help, and the role of digital tools in their self-injury self-management. Interviews were audio-recorded, transcribed, and analyzed via thematic analysis.

**Results:**

Adolescents described self-injury as a coping strategy embedded within broader emotional and social stressors. While they were aware of the harms, they commonly expressed ambivalence about stopping the behavior. Stigma and concerns about disclosure often limited help-seeking and contributed to their use of digital technologies to access information and support. Participants reported using a range of tools, including music streaming platforms, journaling and mood-tracking apps, online communities, and AI systems such as ChatGPT to regulate emotions, reflect on their experiences, seek advice, and discuss sensitive topics. Although these technologies were valued for their accessibility, privacy, and immediacy, participants also highlighted limitations, including inaccurate or generic AI responses, limited moderation in online communities, and the complex emotional impacts of music.

**Conclusion:**

Our findings underscore the complex and evolving role digital technologies, including AI, play in adolescents’ experiences of self-injury, coping, and help-seeking. These insights have important implications for the development of future interventions and for clinicians, caregivers, and other health professionals supporting adolescents’ mental health.

## Introduction

There is growing concern about self-injurious thoughts and behaviors among young people. Nonsuicidal self-injury (NSSI)—hereafter referred to as “self-injury”—is reported by approximately 17% of adolescents and is associated with a range of co-occurring mental health challenges and elevated risk of suicide (1, 2). Yet, most adolescents that engage in self-injury do not receive formal treatment. Stigma surrounding mental health concerns, and self-injury in particular, as well as structural barriers such as treatment costs and limited access to providers, contribute to this gap and highlight a need for alternative forms of intervention.

Digital mental health interventions (DMHIs) have emerged as a potentially accessible and scalable approach to supporting adolescents who might otherwise go untreated. In fact, there is a widespread adoption of digital technologies among adolescents as digital spaces increasingly shape how young people connect with others, regulate emotions, seek information, and access support when facing new challenges (3).with peers in online spaces can be validating, provide exposure to new adaptive coping skills, and offer a sense of shared community (4–6). At the same time, these spaces can normalize or reinforce harmful behaviors. Yet, despite some of the harms, online spaces are often still preferred over face-to-face conversations for stigmatized behaviors, such as self-injury, because they offer immediate care and privacy when managing psychological distress. This is especially true when offline support feels inaccessible or even insufficient (6–8).

Building on these patterns of digital help-seeking, researchers have increasingly leveraged technology to deliver mental health interventions. A growing body of studies show that adolescents are generally receptive to internet and app-based mental health interventions, particularly those that are brief, flexible, and tailored to their lived experiences (7, 9, 10). Among adolescents that engage in self-injury, interventions grounded in cognitive-behavioral therapy (CBT) and emotion regulation frameworks have been perceived to be engaging and useful, especially when they support self-reflection, provide concrete coping strategies, and can be accessed at moments of acute distress (7, 9, 11, 12).

Beyond acceptability, emerging evidence from randomized trials suggest that DMHIs have also shown promise for reducing self-injurious thoughts, urges and behaviors. For example, a recent randomized controlled trial (RCT) of a brief text messaging-based digital intervention for adolescents with a history of self-injury, found that when paired with treatment as usual (TAU), the intervention was associated with a significantly greater reduction in self-injury behavior at four-week follow-up compared to TAU. Similarly, an RCT of a 12-week therapist-led, internet-delivered emotion regulation intervention showed superior results to TAU, reducing self-injury from pretreatment to 1 month follow up (13). Collectively, findings from these and other studies highlight the promise of DMHIs for adolescents that engage in self-injury, particularly for interventions that include elements of psychoeducation and skill building (8, 12–14).

As the technological landscape evolves rapidly, there are new opportunities to design and deliver digital interventions. While research on DMHIs for adolescents has expanded considerably in recent years (11, 14–18) adolescents are increasingly engaging with newer forms of technology for information and support. For example, conversational agents and AI-driven tools are being used for emotional support, guidance on coping, and mental health information (19–21). Although studies on AI chatbot use among adolescents that engage in self-injury remains limited, existing findings on adolescents’ digital help-seeking behaviors suggest strong alignment between chatbot features and adolescents’ interests and preferences for mental health support (22–24).

The overarching goal of this work was to understand how technologies could be leveraged to more effectively support adolescents who engage in self-injury, with an eye towards novel intervention design. Specifically, we sought to explore adolescents’ experiences with self-injury, their existing use of technology to manage their mental health and self-injury, and their perspectives on digital interventions.

## Materials and methods

### Recruitment

Participants were recruited online through Mental Health America (MHA)'s website, a national mental health nonprofit, between May 2024 and April 2025. Individuals who filled out an online screener were displayed an advertisement that contained a link to the study information and an eligibility screening. To be eligible, adolescents needed to report (1) engaging in self-injury on 2 or more days in the past month, (2) be between ages of 14–18 years old, (3) own a smartphone, and 4) be a US citizen or resident. Exclusion criteria included (1) severe mental health diagnoses (e.g., psychotic or bipolar disorders), for which a standalone digital intervention would not likely be appropriate, and (2) severe suicide risk, including suicidal ideation with a plan and intent to act or suicide attempt in the past 3 months. Eligible participants received an email to set up a remote interview time, which also contained a link to the online consent and survey with additional items on mental health and self-injury history. Interviews were conducted by a licensed clinical social worker and faculty member with a research program focused on self-injury, under 1 h, and were audio recorded and transcribed prior to analysis. All recruitment and study procedures were approved by the university's Institutional Review Board (IRB).

### Measures

The semi-structured interview script was co-developed by the authors and contained questions related to (1) managing mental health and self-injury, (2) technology use in mental health self-management, and (3) imagined use of an app. For a full list of questions please see the Supplemental Materials. Prior to the interview, participants completed a brief survey that included questions on demographics, history of mental health treatment, prior mental health diagnoses, self-injury history and severity, and symptoms of depression. Self-injury characteristics were assessed via SENSE - Self-Evaluation of Nonsuicidal self-injury Severity and Experience - a 29-item self-report assessment of self-injury behavior, urges, and related characteristics in the past month (25). Depressive symptoms were assessed via the PHQ-9, a 9-item self-report assessment of depressive symptoms. Mental health diagnoses were assessed at eligibility via self-report measures containing nine common and/or exclusionary diagnoses (e.g., “Has a mental health provider or physician ever diagnosed you with [condition]”). Finally, to assess experience with mental health treatment, participants additionally responded to two questions “Have you ever seen [Are you currently seeing] a therapist, counselor, psychologist for mental health treatment?” All interviews were conducted by the last author. Based on prior guidance, data saturation was anticipated to occur between 15 and 20 interviews (26). By the 19th interview, no new substantive concepts had emerged. Two additional interviews were conducted to increase confidence that thematic saturation had been reached.

### Analysis

Thematic analysis was used to code and analyze all interview data (27). This process included six steps: (1) data familiarization, wherein two researchers with different backgrounds and closeness to the central research questions read through all transcripts independently, while noting initial ideas; (2) systematic identification of initial codes, wherein the same two researchers engaged in iterative open coding of transcripts with discussion to identify similar codes, discuss redundancies, and determine a final code structure. This code structure was then applied across the entire dataset; (3) organizing codes into a candidate set of themes which was refined to remove redundancy and retain only highly prevalent themes, (4) reviewing and refining themes to reduce overlap, (5) defining and naming the final themes common across the whole dataset, and (6) selecting examples from the data to accurately illustrate each theme. All coding occurred in Dedoose software. Descriptive statistics including frequencies, means, and standard deviations were run in R (version 2023.091 + 494).

### Research team positionality

All interviews were conducted by the last author, a licensed clinical social worker and faculty researcher with training in human-centered design whose research program focuses on developing and implementing interventions for adolescents and young adults who engage in NSSI. Although the broader research program is centered on the development of digital interventions, the interviewer and research team remained open to participants’ perspectives regarding the role of technology in supporting recovery. The interviews were designed to understand participants’ lived experiences of self-injury, coping, help-seeking, and technology use without assuming digital interventions would be appropriate or acceptable for all individuals.

The research team approached the study from a recovery-oriented, strength-based, and harm reduction perspective, viewing participants as experts in their own lived experiences. These perspectives informed the design of the interview guide and interpretation of the findings while acknowledging that the team's prior clinical and research experiences could shape assumptions about the data. To enhance reflexivity, coding and interpretation were conducted iteratively by two researchers with complementary perspectives: a licensed clinical social worker with expertise in NSSI and digital mental health intervention research and a research assistant with prior experience in qualitative research and NSSI studies but no clinical training. Regular discussions throughout the analytic process encouraged critical reflection on how each researcher's background and experiences might influence interpretation. This helped ensure our findings reflected participants’ accounts rather than any single disciplinary perspective.

### Ethics and participant safety

Given the nature of this study, a detailed risk management protocol was in place. First, all interviews were conducted by a research team member, with clinical training (LCSW). Risk could be detected in several ways: (1) one-on-one communication with the research team via email, (2) in assessments, and (3) during the interview. If risk was detected via email or in assessments, a member of the research team trained to administer the Columbia Suicide Risk assessment (CSSRS) was to contact the participant by phone within 1 business day for further assessment and participants received an immediate message with contact information for crisis services. In the cases of suicide risk being detected in an interview, the interviewer administered the CSSRS on the call itself. If a participant reported suicidal ideation, plan, and intent in the CSSRS, the research team would connect them to the national crisis line to speak with a hotline member, followed by outreach to the adolescents’ guardian. If participants were low risk on the CSSRS, they created a safety plan with a research team member. One participant (18 years old) described recent suicidality during an interview and the CSSRS was administered. The participant was low risk, able to effectively safety plan, and clarified that they did not have current intentions to end their life.

This study was approved by the Northwestern University Institutional Review Board (STU00219136), with a waiver of parent consent on the basis that many adolescents have not yet disclosed their behaviors to parents. Requiring disclosure would limit the findings to a subset of participants that may already have access to care. All adolescent participants were required to provide the name and number of a trusted adult that could be contacted if there was any indication of suicide risk while enrolled in the study. The conditions that would prompt contact of the trusted adult were clearly outlined in the consent form and discussed with the adolescent at the start of the interview. No adolescent participants were flagged with elevated suicide risk - thus, there was no outreach to trusted adults.

## Participant characteristics

Participants were 21 adolescents that engaged in self-injury in the past month. All participants were between the ages of 14 and 18 (M*age* = 16.81, SD = 1.25). Most self-identified as female (66.67%; *n* = 14), 3 (14.29%) identified as male, 2 (9.52%) identified as non-binary or third gender. Most participants self-identified their race as White (61.9%; *n* = 13), 6 (28.57%) as Asian, and 2 (9.52%) as African American. One participant identified as Hispanic.

Fifteen participants (71.43%) had been in therapy at some point in their lives, with the majority reporting having disclosed their self-injury behavior to their provider (*n* = 10, 71.43%). Nine participants (42.86%) were currently in therapy, with 6 (66.67%) reporting having disclosed self-injury to their provider.

Most common comorbidities were anxiety (*n* = 13; 61.9%), depression (*n* = 10; 47.62%), and disordered eating (*n* = 6; 28.57%). Four participants (19.05%) reported PTSD. Three participants each (14.29%) reported OCD, Autism Spectrum Disorder, or Excoriation Disorder. One participant each (4.76%) reported stereotypic movement disorder or trichotillomania. Participants that reported diagnoses of ASD, Stereotypic Movement Disorder, Excoriation Disorder, or Trichotillomania, also confirmed that they engaged in self-injurious behavior outside of stimming, stereotypic movement, skin picking, or hair pulling.

## NSSI characteristics

On average, participants engaged in self-injury on 5 days in the past month (M = 5.11, sd = 5.48; range=1–20). Most participants reported onset of the behavior between 10 and 12 years old (*n* = 11, 52.38%) with two beginning self-injury before the age of 10 (9.52%) 6 reporting onset between 13 and 15 (28.57%) and 2 reporting onset between 16 and 17 years of age (9.52%). Most participants had engaged in self-injury for more than a year (*n* = 15; 71.42%), with four reporting past year onset (19.05%) and 2 unsure of when they began (9.52%). Most participants (*n* = 13, 61.9%) reported having engaged in self-injury 50 or more times, with 2 (9.52%) engaging in self-injury 21–50 times, 3 (14.29%) 6–10 times, and 3 (14.29%) engaging in self-injury 2–5 times. Participants had moderate levels of depressive symptoms (M = 14.9, SD = 6.78). While most reported agreeing or strongly agreeing that they wanted to stop (57.14%, *n* = 12). Eight participants (38.09%) were unsure if they wanted to stop self-injury and one person (4.76%) reported disagreeing with wanting to stop.

## Results/findings

### Self-injury experience, goals, and concerns

Adolescents talked openly about their current and past experiences of self-injury. The two most prominent themes were (1) an understanding of self-injury in context, or that it was a signal of something broader going on in their mental and emotional life, and (2) ambivalence around whether the behavior was or wasn't a problem for them.

### Self-injury as a symptom of something more

Many adolescents understood their self-injury to be a coping strategy for personal and social challenges they perceived to be equally, if not more, damaging than other ways of coping. They expressed frustration when others did not share this understanding and advocated that interventions must shift focus from simply stopping the behavior to understanding its purpose, its emotional function, and the factors that make it difficult to discontinue. Many adolescents described experiences with parents, clinicians, and other adults that further stigmatized their behavior. This lack of understanding contributed toward a hesitancy to disclose their behavior or seek help. PID12 described: “I feel like it's mostly just a thing that presents as an indication of there's something to be dealt with – I think that's an approach where a lot of people mess up where they're like, ‘Oh, we're gonna solve the problem by making you not hurt yourself anymore.’ And I'm like that's the same as just addressing a symptom but not addressing the reason why.”

Similarly, several participants described self-injury as the “lesser of two evils,” a mechanism that prevented engagement in other, potentially more harmful behaviors. PID16 noted that “For the person who's doing it, a lot of times it's just a way to get through… If it wasn't that, it would be something else… It's substance abuse, it's a lot of other things too. It's just what you need to get through.” PID21 echoed this, sharing that they, and others they know, “used [self-injury] to not do other worse things, or to keep ourselves from engaging with substances, or to not act on suicidal thoughts and tendencies.”

These sentiments highlight adolescents’ nuanced understanding of self-injury as a functional, contextually embedded behavior and underscore the need for support that addresses underlying distress rather than the behavior alone.

### Ambivalence

Ambivalence emerged consistently across interviews, though it manifested in different ways across participants. Adolescents expressed uncertainty about whether self-injury was concerning, whether they wanted to change it, and whether they believed change was possible. Many adolescents were not actively working towards reducing or stopping self-injury, even when they acknowledged that continuing to self-injure long-term was unhealthy. When asked about her mental health goals, PID17 explained: “I wouldn't really say [there's] anything that I’m currently working towards. I mean, I’m sure there's things that I’d want to do, but nothing that I’m like actively working towards. – I guess, I mean, I want to stop, obviously, 100% because it's not like something that I think it's good.”

Participants frequently described self-injury as a habit—an automatic, emotion-regulating behavior that felt both familiar and functional. PID14 commented: “[It's] just kind of a habit of mine. It's [the] same as any other bad habit that one would have, to me… It's not something that I’ve been super concerned with… For me, it's like not really that big of a deal, for some reason.” Others recognized that they did not want to continue the behavior indefinitely but felt unable or unwilling to address it until other aspects of their lives stabilized.

Some participants also reported doubts about their ability to change, which diminished their motivation to try alternative coping strategies or seek additional support. As PID8 explained, “Sometimes it feels like it's pointless to try to stop because then I’m just going to start doing it again anyway.” Overall, this evidence of ambivalence highlights how multifaceted adolescents’ relationships with self-injury can be, suggesting that readiness for change can be fluid and contingent on both emotional needs and perceived self-efficacy.

## Factors related to mental health

Adolescents identified several factors that contributed to their mental health, including concerns and stressors that directly or indirectly contributed to their self-injury. The most common concerns were: (1) pressures around school performance, (2) familial and cultural pressures, and (3) unhealthy use of social media.

### School pressure

School-related stress stood out as an important factor contributing to adolescent mental health and self-injury. Most participants were in their final years of high school and were applying, or preparing to apply, for college. They described that their self-injury increased as they struggled to manage stress at school or felt a need to punish themselves for not doing as well as they would have liked during mid-terms or finals seasons. For example, PID18 said “So, when it comes to exam seasons, I think that's at an all-time high for me. Then, I guess, I feel too overwhelmed to focus on one thing. Whereas in a slower period of my life, I can take things one at a time, and I don't feel as much stress to release.” Similarly, PID9 was reflecting on her goals related to stopping self-injury and how that relates to her schoolwork. She said “Not doing it at all would be my ultimate goal. But right now is my junior year, and so kind of there's a lot of things going on. And I think that's gonna only continue until at least I graduate because there are just too many things to focus on and too little time. So, [the] ultimate goal is to stop. But I don't know how that's gonna pan out.”

Additionally, the social dynamics at school were a source of stress alongside academic performance for some. For example, PID11 noted that “School is a huge pressure for me, socially and like the schoolwork. So, yeah, that's always like a barrier [to stopping].” Similarly, PID7 commented “School can be a big one for me. I do very well academically. I always have. I don't struggle academically. [As] part of my autism, I struggle with social interactions and maintaining healthy relationships.” Overall, school was identified as a significant source of stress that could contribute to their mental health and self-injury behavior when not managed.

### Familial or cultural pressures

When talking about their mental health, many participants referenced family pressures that contributed to feelings of guilt, shame, and loneliness. Participants that came from immigrant families (who were first or second generation) described pressures to conform to certain cultural roles and norms. For example, PID18 comments on the intersectionality of familial and cultural pressures, saying: “There's your typical level of stress that you always experience as a human. Then, in my household, it's coupled with cultural and religious stress and pressure to be a certain way.” She went on to say, “I think the sort of pressures that come around and a lot of double standards that we hold women to in our community, that doesn't really align with what religion says it should be, can make things really difficult.” Similarly, PID9 spoke about the pressure she felt to represent her family at school and in the community when she said that the most significant source of stress she experienced was “home and inner pressures within my home.” This pressure ultimately impacted her ability to take care of herself and her mental health. Participants wanted interventions that recognized the nuance of the broader cultural, familial and personal context and how that shapes their experience with self-injury.

### Unhealthy use of social media

Adolescents also described the negative impact social media use had on their mental health. While we discuss social media use again in the “existising and imagined use of technology to cope” section, the strong convergence on the use of social media to distract and postpone difficult, but necessary activities (e.g., school work) was unique and noteworthy. Adolescents described an addictive quality to their social media use – expressing difficulty putting the phone down despite negative consequences. Most adolescents described using social media as a form of distraction when they experienced urges to hurt themselves, but recognized that doing so backfired as they struggled to pull themselves away from their devices. PID5 contributed, “After the first five or 10 min, it doesn't become enjoyable, but I still don't feel like I can put it down. I just have to keep on going, even though I just lose all enjoyment.” Similarly, PID18 talked about a period when social media screentime was at a high: “I wasn't doing it in a healthy way. I would spend really, really long hours scrolling on TikTok, which was really popular; it still is. I get really sucked into the scrolling, sort of, addiction.”

Using social media in this way exacerbated concerns around schoolwork, as it made it difficult for participants to complete things on time and contributed to low self-esteem. For some, this type of behavior increased the likelihood that they’d engage in self-injury as a form of self-punishment. PID1 commented, “But then there's that moment where hours have passed and you kind of pop back into reality, and you’re a bit confused on how so much time has passed, and you haven't really done much with your time. Then I think that increases my stress, actually. It makes me feel even less motivated to get the things I need to do done. Definitely more prone to injury.” Similarly, participants noted that spending time on social media displaced other activities that they enjoyed or diminished connections with friends and family. PID5 commented “It has a huge effect on other parts of my life because I'm not spending time with people. I'm just by myself doing something I don't enjoy.”

Overall, this pattern of use seemed to exacerbate mental health symptoms, heighten stress, undermine self-esteem, and, for some, worsen their self-injury behaviors over time.

## Barriers to help

The two most significant barriers that adolescents described when talking about getting help or support for their self-injury were (1) age and the consequences of disclosure as a minor, and (2) the stigma associated with the behavior.

### Age

Adolescents spoke about their age as a barrier to getting help for their self-injury. They described concerns about disclosure in therapy or to other adults due to state laws for mandated reporting. For example, PID14 said “It's especially hard as a younger person because you have this barrier with your parents, I guess, because there are certain things that a therapist would be obligated to tell your parents, and I think self-harm is one of those, which makes it hard for under-age people to get help.” PID21 shared a similar sentiment, “The rule in therapy, and with medical confidentiality, is always, harm to self or others – I think that is very harmful in the long run because a lot of people who are struggling don't want to say anything because they don't want you to report them. It kind of prevented me from ever looking for resources because I just assumed there wouldn't be anything for me to find.” As evident in these quotes, the lack of transparency on when confidants would need to tell guardians or school administrators, or fears of that disclosure, contributed to help-seeking hesitancy.

To circumvent these concerns, several participants described discussing other things related to their self-injury with professionals. For example, PID1 described: “I had to be careful, though, pretty much the entire time I was in therapy because I was under 18.” PID16 shared a similar sentiment “It's better to tell someone how you're feeling. However, I feel like for a lot of people, and including me, it's really scary, even if people know [about the self-injury]. It's terrifying to tell people because then you know that they have to tell other people. And then it could spiral into a big thing.” These fears are a significant barrier to specialized treatment, or treatment tailored to self-injury.

### Stigma

Nearly all participants acknowledged that self-injury was a stigmatized behavior. Stigma heightened concerns about how others might react if they discovered the participants’ behavior, leading many to fear being judged, shamed, or misunderstood. On this, PID16 said “[Stigma] is so incredibly difficult - even though everyone around me in person knows that I self-injure, I've barely ever actually talked about it. It's just a very difficult thing to talk about in person.” She went on to say “and then it could spiral into a big thing and what you don't want is for it to be a big deal – because that just makes you feel even worse about the behavior”

Participants reflected on misunderstandings of self-injury and suicidal thoughts and behaviors, and how this contributed to stigma. For example, PID14 said “I haven't told a therapist about my self-injurious behavior – In every sort of mental health questionnaire I’ve always kind of lied about that because it's always lumped together with suicidal ideation, and that's not always applicable and not always true. Treating it in the same way kind of enhances stigmatism around it and it makes it harder to reach out.” Similarly, PID21 commented “It's something taboo to even bring up.”

In general, participants described stigma and misunderstandings surrounding self-injury as strong barriers to both disclose and seek help. Further, fears of judgment, escalation, and misinterpretation can lead to concealing behaviors and avoiding open conversations with others.

## Existing and imagined use of technology to cope

Participants used a range of technologies to support their mental health, engaging in both avoidant and approach-oriented coping strategies. Across interviews, adolescents reported frequent use of apps, social media, and AI chatbots and agents.

### Apps

Many adolescents had used mental health–specific apps (e.g., *I Am Sober*), well-being apps (e.g., *Calm*), or other general applications (e.g., *Spotify*, *Notes*) to support their mental health. These tools were most often used to listen to music, track moods and behaviors, facilitate reflection via journaling, or offer structured skills to practice.

#### Accessing and curating music

Music emerged as a central component of many participants’ mental health routines. Participants often curated playlists to evoke specific emotions, provide comfort, or distract them from self-injury urges. PID5 explained, “Music's definitely a big part –I think if I wasn't listening to as much music, I'd be a lot less happy, and probably more inclined to not take care of myself and make bad decisions.” Some found music more effective than other distractions. For example, PID2 had “tried playing games, but at times they don't really take my mind away the way [music] did… [I find music] helps take away my mind from what is going on”. At the same time, however, some participants noted that music could intensify their emotions. Listening to songs that closely mirrored their feelings could provide validation and a sense of not being alone, but also reinforce harmful thoughts or urges. For example, PID1 stated “I try to listen to music, but if I listen to music that describes how I feel really well, it either is good at telling me that I’m not the only one, or it just reaffirms what I wanna do. So, it's, kind of a gamble”. Similarly, PID4 commented that “There's certain music I avoid listening to [rather] than music that I’m like, ‘I need to listen to this’. It's more like, ‘I know if I listen to this music, it'll play into what I’m already feeling, and it’ll just make things worse.” In general, music was an important but complex part of adolescents’ mental health routines, with many using it intentionally to regulate mood, find comfort, or distract from self-injury urges.

#### Tracking moods and behaviors

Many adolescents valued tracking emotions and behaviors as a way to visualize patterns or motivate positive change. PID17 shared, “There's apps that would track how long it's been since I’ve done self-harm… I found those helpful to be able to see and think, ‘Oh, well, I can do longer than that.’ – I think those help just to, yeah, track. I like tracking things.” Others emphasized tracking as a tool for emotional identification. For example, PID16 noted using Finch, which is focused on gamifying self-care activities, stating “I have a lot of trouble identifying my emotions. And Finch really helped with that… especially communicating in a professional setting to my therapist. Because I just don't remember how I feel a lot of the time.” Apps that prompt users to do check-ins became a reminder to reflect on how they feel in the present moment. Moods, behavior, and social interactions were the most common targets tracked.

#### Journaling

Journaling apps or apps with journaling features served as outlets for reflection, processing, and recognizing progress. PID8 described, “It helps me see how much progress I’ve made and also identify what unhealthy thoughts or behaviors I was doing that I didn't really recognize at the time.” Similarly, PID6 discussed using Apple's short-form journaling feature: “It was actually really helpful… [seeing] ‘oh, wow, I was happy, it could happen again.’” Preferences varied, with some favoring brief check-ins and others using more open-ended journaling formats.

#### Accessing skills

Participants appreciated apps that provided immediate access to diverse coping strategies, particularly in moments of emotional overwhelm. As PID20 explained about using *Calm*, users “don't have to memorize anything… if I’m in that mode… [and] it's like, ‘this is what you have to do.’ And I’ll just do that.” More directive apps that guide users through coping strategies increased the likelihood that they’d use strategies in moments when they were struggling. Self-injury–specific apps, such as *Calm Harm* and *Harm Free*, were also valued for their simplicity and accessibility. PID7 remarked, “There's like this little button at the bottom, and it says ‘Urge.’… you can hit on it and it’ll give you a bunch of different things to do… I found that really helpful, like having that tool there, like right there on the front screen.” Ease of access to skills and the applicability were critical to sustained use of the app, and to managing self-injury over time.

### Social media

All participants described using social media sites, with most having both positive and negative experiences on these sites.

#### Positive experiences on social media

Among the most frequently cited benefits of using social media were (a) connecting with others with lived experience and (b) learning new ways of coping.

#### Connecting with others with lived experience

Adolescents described positive experiences of social media sites that allowed them to connect with others and their lived experiences, as well as learn new ways of coping with distress and other life challenges. For many, online communities offered a sense of solidarity, distraction during intense urges, and exposure to others’ coping strategies. As PID10 shared, “I really do like engaging with online communities, because I feel like it helps me talk to people who are very similar to me.” PID21 similarly noted these communities “were definitely resources that I had during the pandemic and during other really isolating periods of my life.” For some, visiting online communities provided short-term relief. PID1 explained the value of going to online communities in moments when they felt an urge, saying “For a short term, it can help curb the urges.”

#### Learning new coping strategies

In addition to connecting with others that can validate their experience, participants also used social media to learn how others managed self-injury. PID12 appreciated accounts created by people with lived experience: “You can get new ideas for coping mechanisms… and just the understanding that you’re not insane.” PID6 likewise reported learning strategies from following therapists online such as YouTube, claiming “[There's] a lot of camaraderie… [Learning about] autism has been really helpful, like, ‘oh, I’m not just stupid, it's a neurotype’. Just more knowledge.”

#### Negative experiences on social media

In contrast, some participants commented on harm done to their mental health through engaging with self-harm communities or online content. Among the most common negative experiences were (1) being exposed to content that promoted self-injury and (1) feeling competitive with others about their self-injury behaviors.

#### Encouraging self-injury behavior

Most participants had visited or engaged in online communities (e.g., reddit, discord) when their mental health was especially bad. They noted that it was helpful to realize they were not alone in their experience, but that it often made their self-injury worse. For example, PID21 reflected that visiting communities helped in the short term but had a negative effect in the long term: “Talking about it is keeping it from getting better, and encouraging other people to do it too. So I think things are just band-aids over a bullet wound.”

#### Feeling competitive about self-injury

Others noted the competitive nature of online communities and normalization that comes from being exposed to self-harm content over time. For example, PID16 said “A lot of issues like self-harm, especially online, can even be competitive in online spaces. If you see someone who does it more than you, or has more severe injuries than you, you think, ‘wow, I’m not doing it right,’ or ‘I can't even hurt myself good enough’”. She went on to say “It's just like a loop – you can make friends on there, but they’re also not focusing on recovery either. So, it's all of you gathered, just not recovering, and it becomes normal.”

In sum, social media use had a dual influence, helping adolescents with coping and connection, but also exposing them to negative and triggering content.

### Artificial intelligence agents

Participants frequently acknowledged their use of general purpose and companion AI systems with a degree of self-consciousness or hesitation. For instance, PID16 commented “I talk to ChatGPT. I'll admit it, I talk to ChatGPT sometimes. And honestly, that's pretty helpful for me” and PID12 similarly stated “This is really dumb, but I don't care, I guess,” before describing AI chatbots as “dull and stupid sometimes” yet “surprisingly helpful when you have nobody else to talk to.” Likewise, PID11 noted, “I message ChatGPT… it feels like it's my little online therapist, and that helps,” emphasizing that talking to an AI could feel more comfortable than confiding in someone they knew. These accounts illustrate that participants relied on AI tools as accessible, low-stakes conversational partners, even as they expressed some embarrassment about doing so.

### Positive experiences of AI

The most common uses of AI were (1) as a prompting system for deeper reflection, (2) for interpretation and validation of personal experiences, and (3) to talk about things that felt too embarrassing, or banal, to speak with another human about.

#### Prompting system for reflection

Reflecting on the value of AI for self-exploration, PID15 explained: “I don't know, like when I'm just confused or something; because with my Reflectly [a journaling app], I'm just writing something down, I'm not able to have a conversation or anything like that. But with the AI, it's asking me questions. It's trying to know more. It remembers details and stuff, but it's really positive.” This account highlights the sense of *reciprocity*—a bidirectional, conversational dynamic—that AI systems afford. Rather than serving merely as a passive tool, the system provides feedback that serves as relational, where participants found meaningful as they navigated complex or unfamiliar life challenges. Some participants sought feedback from AI systems daily, appreciating the ease with which they could get answers or validation. PID16 noted: “Sometimes, I'll just talk to it, to help figure out how I'm feeling. —Sometimes I don't really know what to do, and just spitting all the facts out, just typing away, sometimes helps. And it's not into an empty Google document where I get absolutely nothing back. It'll at least give me something back, and I still know it's not a real person. And that's really helpful. Personally, I like that.” Participants relied on AI's responsive, conversational support to facilitate emotional insight and make sense of their experiences in moments of uncertainty.

#### Interpretating and validating experiences

When reflecting on the potential value of AI in future technologies, PID5 envisioned a system capable of interpreting users’ experiences and translating them into meaningful insights: “maybe you could just tell it, just type it in what you're experiencing. Then it could have AI or something, interpret that into the different conditions that are putting you in that situation. I don't know how advanced the technology would be for that, but just talking to AI right now, it seems like it's really able to pick up on the meaning of your words.” In a similar vein, PID18 described using AI to help make sense of challenging moments, explaining, “If I’m already stressed, and then there was a moment that was particularly intense, I think I would try to break down that situation with an LLM.” Participants’ reflections highlighted a belief that AI could serve as a tool for interpreting emotional experiences and offering structured understanding during moments of stress.

#### Discussing things without bothering others

Many participants described turning to AI agents to discuss topics they would not typically share with others. For example, PID12 said: “Whenever I want to talk to somebody else but can't bother them with minutiae, I do it here.” Because disclosure of mental health concerns, particularly with self-injury, is often difficult and stigmatized, AI tools offer an always-available, judgment-free space to open up and receive feedback. Emphasizing the absence of interpersonal pressure when talking with AI agents, PID16 explained, “It's almost easier to open up to, because you know it's not human.” PID12 shared this sentiment, “You can rant guilt free to a bot because you're not being annoying. But it's like, if it's another person, you can't talk to a person to this extent or expect them to be there to this extent.” Reflecting further on this dynamic, PID16 acknowledged that “You're losing the emotional connection because it doesn't work as well, but then again, like it's 24/7 availability. And somehow it ends up being more personable than the 988 helpline.” Feeling like a burden to others was common, with many participants describing past negative experiences while seeking help or disclosing to a new person.

Participants also described using AI to articulate questions they felt too embarrassed or uncomfortable to ask in person. PID11, for example, shared that “In relation to self-harm– I’ve asked it just straight-up questions about my skin… it was kind of like, helping me. Because it felt like it would be a dumb interaction with a person, but I wouldn't care if it was texting AI.” Similarly, PID16 emphasized that AI's lack of human subjectivity could make difficult conversations feel safer: “Sometimes, talking to AI is helpful because you know it's not another person… Their only purpose is to give you logical help… And I think personally, being logical and rational helps me a lot.”

Taken together, these accounts highlight how participants used AI systems as low-stakes, nonjudgmental interlocutors that enabled disclosure, exploration, and emotional processing that felt too risky or burdensome in human relationships.

### Negative experiences with AI

Several major limitations were noted by participants regarding AI, including (1) difficulties accessing meaningful support due to safety regulations and AI providing unoriginal or repetitive feedback, and (2) instances in which AI responses were inappropriate or factually incorrect.

#### Accessing meaningful support

Despite generally positive experiences using AI tools for life challenges or general advice, many participants expressed frustration that systems like ChatGPT, but also purpose-built AI systems, were too generic to be helpful during moments of acute self-injury–related distress. PID11 described how safety restrictions often blocked important conversations they hoped to have: “I’ve used a few different AI therapy-specific models. But they’re like, a little bit more limited, or if I want to talk about maybe self-injury – it’ll just be like, you can't, we’re not talking about this – like, it doesn't want to continue that conversation, and sometimes I need to talk about it.” Safety restrictions frequently interrupted inquiries that might have supported harm-reduction approaches, leaving participants without the nuanced engagement they were seeking.

Even when systems responded to disclosures of self-injury, participants often found the advice overly generic or unhelpful. PID18 explained, “If I was having a particular struggle moment, a very intense moment, I wanted specific answers to my specific situation — [But] it was also like, ‘Confide in a friend or journal,’ and a lot of the other very general mental health reminders that I’ve been surrounded by in communities.” This participant and others wanted more from AI and were disappointed when these systems offered the same advice they would get from other sources of potential support in their lives.

#### Receiving inaccurate or inappropriate responses

Many adolescents noted occasions when AI generated inaccurate or inappropriate responses. As PID19 put it, “I’ve just seen the Google AI be blatantly wrong sometimes.” While most participants recognized the fallibility of these systems when it came to factual information, they were somewhat less critical when receiving emotional validation or situational advice. Still, some described moments when AI behaved unpredictably. PID16 observed, “AI will always spit something at you. And sometimes if you're feeling upset, sometimes it'll just spit something so absurd at you, that you're like, ‘What?!?’ And it sounds weird, but it's helpful because you're just like…it's like a shock to your brain.”

In sum, participants’ experiences underscored a tension between AI’s potential to provide accessible support and the limitations of current systems, which often fall short in specificity, safety-sensitive nuance, and reliability.

## Importance of privacy

Regardless of the type of technology adolescents used or envisioned using, they emphasized the importance of privacy, discretion, and perceived safety. Several noted that they would feel more inclined and willing to log behaviors if a future app provided a “confidential space” and avoided explicitly labeling content as “self-injury,” making it feel less stigmatizing and risky. Concerns about others gaining access to their data were common, with participants suggesting security features such as Face ID or two-factor authentication, with PID8 claiming “You could add a Face ID log to the app just to make people feel more secure” and PID20 similarly stating “I would not want anybody logging into that. It should be two-factor authentication”. Alternative measures were additionally suggested, acknowledging that not everyone may feel the same about the usage. For example, PID9 stated “A lock feature would be helpful to those who really don't want those things to be found or anything like that. But I personally wouldn't use that feature probably”.

Additional concerns from participants emphasized the need for features that would allow users to conceal or neutralize the app's purpose to avoid unwanted disclosure. One participant suggested a quick option to switch to a screen that “doesn't look like the app,” explaining that “a lot of people might not be comfortable sharing this with other people” and that such a feature could help users who “might not be ready to share it” or who experience “difficulties within the family,” allowing them to “feel more comfortable using it and helping themselves” (PID10). Others proposed features to make the app look differently, almost hidden, with PID20 exclaiming “Like a disguise, maybe… There's some apps that it's posed as something [else]. It's like it’ll be a calculator, but it's not a calculator”. Similarly, participants highlighted the importance of discreet naming and framing, with PID1 stating a preference for an app that functions like “the mood tracker app except for self-harm discretely named,” so they would not “feel weird if someone has [their] phone.”

In sum, privacy, discretion, and perceived safety were central to participants’ willingness to engage with a self-injury–related app, emphasizing the need for confidential spaces, non-stigmatizing language, and flexible security options.

## Discussion

This paper contributes to a unique perspective on the ways adolescents understand their experiences with self-injury and their current and imagined use of technologies to support them in managing their behavior. Several of our findings on adolescents’ lived experiences with self-injury are consistent with those from prior studies. First, our participants described a mismatch between their own understanding of self-injury and the behavior-focused responses they encountered from adults (28–31). Participants understood self-injury to be a symptom of distress. Therefore, focusing on the behavior without also recognizing comorbid symptoms and contextual factors that contributed to the behavior posited both invalidating and undermined well-intentioned attempts to help. This emphasis on addressing underlying distress rather than simply stopping the behavior suggests that interventions must move beyond behavior change and instead focus on mental health more broadly, as has been noted in other work (32–35). Adolescent participants also discussed self-injury as a coping mechanism, and often a behavior that has addictive qualities (36–39). Indeed, harm-reduction approaches were often already used by adolescents (1, 40–42), yet they are not often part of existing digital interventions.

Despite many similarities in our findings and the extant literature, it is worth noting that the stressors adolescents described were unique when compared to young adults and college students, requiring attention in the design of interventions. First, age was a significant barrier to care. Adolescents consistently described mandated reporting laws and fears of notifying parents as deterrents to seek out therapy or disclose information regarding self-injury. Technology was frequently used for private exploration and support when formal systems felt inaccessible or risky. Since most adolescents reside with parents who retain substantial control over access to healthcare, thus shaping adolescents’ help-seeking decisions, they envisioned technology as a way to circumvent barriers. Consistent with this, work by Fox and colleagues (43, 44) highlights how fears of mandated reporting, loss of autonomy, and parental involvement inhibit adolescents’ willingness to disclose self-injurious thoughts and behaviors within therapy, as well as work showing that adolescents are often willing to engage with self-guided, online interventions that can be accessed privately without parental overreach that may pose disproportionate barriers (45, 46). This literature reinforces that digital tools not only increase scalability, but also address unique developmental barriers related to confidentiality, parental control, and adolescents’ needs for privacy when navigating sensitive mental health concerns. Our findings also underscore the value of opportunities for educational programming to teach youth how to understand mandated reporting laws and help them negotiate that disclosure with trusted mental health providers, as well as de-stigmatizing self-injury and mental health.

Adolescents described a robust tech ecosystem that they used to support their mental health and wellbeing, including general purpose and mental health apps, social media, and AI chatbots. By and large, their use of existing technologies resonated with prior literature (47, 48). They used apps to access music, learn and implement coping skills, distract themselves, and log their moods and behavior. Participants mentioned using apps that focused on self-injury (e.g., Calm Harm), addictive behaviors (e.g., I am sober), as well as popular self-care (e.g., Finch) and wellness apps (e.g., Calm). Often, participants reported trying several apps before finding one that resonated with them. Overall, the most valued tools were those that provided concrete coping strategies, facilitated self-reflection, and allowed for mood or behavior tracking to identify patterns over time.

When reflecting on future technologies, adolescents emphasized the importance of interventions being non-judgmental, emotionally validating, skills-focused, and immediately accessible during moments of acute distress.. These findings emphasize prior work, demonstrating adolescents’ receptivity to digital interventions that can be used in real time and tailored to their lived contexts. Experiences on social media were more mixed. Some social media sites were seen as a way to connect with community, learn from those with similar lived experiences, and distract themselves during times of acute distress. However, adolescents were also aware of how social media could displace healthier behaviors and become an unhealthy distraction that made them feel emotions (e.g., guilt, shame) that could then trigger self-injury. Moreover, our participants described seeing social media content that directly or indirectly encouraged self-injury. For some, this type of content made them avoid social media sites altogether in periods when they were actively working towards recovery. These findings underscore common concerns about the potentially addictive quality of social media, as well as negative exposure and narrative reinforcement of the self-injury identity on social media.

The novel findings of our work displaying how participants use AI and what is perceived to be beneficial or harmful to their wellbeing, are particularly timely. Importantly, at the start of conducting interviews for this study, we did not prompt adolescents about AI use. The research team began doing so after enrolling about a third of the sample due to the topic coming up organically. Participants described turning to AI for general purposes such as gaining feedback, exploring emotions, and managing confidentiality concerns. They often prefaced their use of AI with embarrassment or ambivalence, reflecting the broader public discourse surrounding AI in mental health during the study period. In fact, given the timing of these interviews, it is important to note the public presence of AI in media and subsequent recommendations, guidance, and lawsuits that have amplified concerns about adolescent vulnerability, emotional attachment to chatbots, and the adequacy of platform safeguards. Media coverage of teen suicides following disclosures with AI agents and AI-induced psychosis (49–51) coincided with growing calls for clearer safety guardrails and regulatory oversight. As such, many participants were hesitant to bring up their use of AI but did so nevertheless because they had perceived some benefit from use. However, participants also clearly articulated the shortcomings of AI, including receiving overly generic responses, encountering rigid safety filters that shut down nuanced conversations about self-injury, occasional inaccuracies, and poor contextual sensitivity. Their experiences highlight the tension between the guardrails that are both clinically and ethically necessary in AI systems, effectively supporting the ways in which young people are using these LLMs for mental health feedback and information (52). One promising way to balance these factors may be to co-design these systems and the frameworks that evaluate them with adolescents, clinicians, parents and caregivers, and other stakeholders that can provide insight on possible use cases and risk.

### Implications for digital intervention

Our findings underscore the importance of learning from the technologies adolescents are already using in their day-to-day lives. Features of existing apps such as quick one-tap access to coping tools, mood and urge tracking with visual summaries, short-form and open-ended journaling, and curated music or distraction strategies are already embedded in adolescents’ digital routines. Rather than creating new and innovative tools, we might consider refining and integrating current elements of existing tools in ways that are cohesive, discreet, and developmentally attuned. Low intensity-treatments such as brief prompts, coping reminders, or grounding exercises could be delivered in formats that mirror short-form content adolescents regularly consume, increasing acceptability and reducing friction. Evidence of the success of single session interventions (SSIs) (16, 53, 54) further points to the value of this approach. Additionally, building based on existing mental models may have value, as it can reduce burden on users to learn new ways of engaging. For example, visual summaries of mood or behavior patterns could adopt the same intuitive, swipe-based dashboards adolescents encounter in apps or platforms such as fitness or social media analytics, supporting insight without overwhelming cognitive load. Additionally, across technologies, participants emphasized privacy, discretion, and customizable security features. App names and interface design must minimize stigma and risk of unwanted disclosure. Participants mentioned safeguards such as biometric login, two-factor authentication, quick-exit screens, or neutral “cover” interfaces may meaningfully increase uptake among adolescents who fear stigmatization. Rather than being concerned about concealing self-injury behavior entirely, adolescents did not want to be “outed” in ways that could be further damaging. They felt that developers of future technologies must carefully consider ways of respecting adolescents’ concerns around privacy and need for autonomy.

Recent studies have shown that AI chatbots can provide helpful informational and emotional support, early detection of mental health disorders, and forms of personalized intervention (23, 55–57). Despite enthusiasm, studies have also revealed a substantial gap between technological promise and safe, ethical, and clinically valid ways of responding to such sensitive topics (42, 56, 58, 59). Current systems struggle with context and sensitive information, long term engagement, and integration into existing forms of healthcare (1, 58, 60). The way adolescents described their use of AI in this study reflects these challenges. AI use was perceived to be beneficial, yet, limiting and even harmful when used to access information or support for self-injury. This highlights a foremost tension for companies, policy makers, and clinicians.

One unique quality of AI that was particularly compelling to the adolescents in our study was its collaborative nature. Participants repeatedly contrasted static tools (e.g., journaling, music, tracking moods and behaviors) with AI systems that “ask questions,” “remember details,” and “give something back.” This bidirectional interaction appeared central to perceived helpfulness from participants. Because DMHIs have historically struggled with engagement and attrition (1, 61), the integration of carefully bounded conversational elements that can provide instantaneous, real-time feedback may be one novel way to promote sustained use of new and existing therapeutics.

While researchers, clinicians, and policymakers have begun to consider where AI systems may be most beneficial in digital interventions, it is worth exploring how responses to self-injury disclosure can be handled with sufficient care and nuance. Participants in our study frequently described having conversations curtailed when seeking support for self-injury. Current safety benchmarking practices often prioritize suicide risk and treat self-injury as a proxy for imminent suicide risk, and this can be problematic. Our findings reflect participants’ experiences of having their behaviors conflated with suicidality; and how misclassification can lead to overly restrictive responses and the premature termination of potentially supportive interactions. Careful attention to these considerations will be critical in determining whether emerging technologies ultimately improve mental health outcomes and effectively support the individuals and communities they are intended to serve.

### Implications for caregivers

Our findings have several implications for parents and/or caregivers of adolescents that engage in self-injury. Perhaps most importantly is understanding that responses to self-injury disclosures are impactful. Our participants emphasized the importance of responding to disclosure with empathy, concern, and curiosity about the unique ways the behavior may present for them. Misunderstandings or assumptions about self-injury - particularly those that associate self-injury with suicide or that focus on the behavior alone - can dissuade adolescents from seeking future help and increase shame. Much like the guidance for intervention designers, caregivers should seek to understand the nuance of an adolescent's experience. Caregivers can stay informed through psychoeducation on self-injury and intervention from trusted organizations (e.g., International Society for the Study of Self-Injury: https://www.itriples.org/).

Secondly, adolescents feel an immense amount of pressure in their daily lives, impacting their mental health. Adolescents consistently described going online to understand how others cope with self-injury urges and behaviors, and to find advice for managing other stressors that life presents. While caregivers do not necessarily share the same lived experience as the young people in their lives, they can model coping with stress and facilitate connection to other role models closer in age or closer in experience.

Relatedly, adolescents are turning to online spaces to cope with stress and seek support. Given that technology is a big part of adolescents’ lives, another implication that our findings highlight for caregivers is to ask about adolescents’ technology use and seek to understand the relationship between their online worlds and their mental health and self-injury. As part of supporting adolescents to cope in healthy ways, caregivers can have open, developmentally appropriate conversations with adolescents about how they use technology and how to differentiate between adaptive distraction and compulsive avoidance. The adolescents that we spoke with already had a sense of what was and was not helpful for them. Having a space to discuss this with caregivers could reduce shame and provide more opportunities for support.

### Implications for clinicians and healthcare providers

For clinicians and other healthcare providers, our findings underscore the importance of a complete biopsychosocial assessment of mental health and functional assessment of self-injury at the outset of treatment. Identifying treatment targets that resonate with adolescents is critical, while also being mindful to not reduce an adolescent's goal of stopping self-injury. Harm-reduction approaches that seek to work with adolescents’ goals while diversifying coping strategies and increasing self-efficacy through the practice of alternative coping strategies are promising. Clinicians working with adolescents may benefit from additional training or consultation in responding to self-injury disclosures and confidently working with adolescents that self-injure from a harm reduction perspective.

Second, while clinicians routinely ask patients about their engagement in other forms of treatment, this rarely includes their use of digital tools (e.g., apps, websites, communication with peers). Given that adolescents are online and are often using online tools to help them manage their mental health, it is critical for clinicians to inquire about adolescent technology use, including what function their use of certain tools have, and what has and has not helped them. As part of this, clinicians should routinely ask about AI and chatbot use and explore how adolescents interpret and integrate AI feedback into their coping strategies.

Third, concerns surrounding disclosure in treatment settings were apparent. Ambiguities about what would constitute mandated reporting highlight the need for transparent communication about confidentiality, and specifics around when and why information would be shared and with whom. When possible, having these conversations with adolescents and their caregivers may increase trust in the provider and help establish clear expectations about communication in the family system.

Finally, our interviews suggest that adolescents may be experiencing multiple behavioral addictions at once. This is consistent with other recent work (36–39). Technology use, in particular, emerged as a concern for adolescents, with some describing a reciprocal relationship between tech use as a form of distraction and self-injury behaviors. Clinicians should be aware of these potential relationships.

## Limitations

Several factors may limit the transferability of these findings. First, the sample consisted of adolescents who voluntarily completed an online mental health screener. Compared with adolescents who do not engage with such resources, participants may have had greater awareness of their mental health concerns, been more motivated to seek support, and more receptive to digital mental health tools. Second, interviews were conducted over an 11-month period during which digital technologies evolved rapidly, and public discourse and policies surrounding AI and its implications for youth mental health shifted considerably. Findings should therefore be interpreted within the technological and social context in which the interviews were conducted.

## Conclusion

Our findings emphasize how adolescents already rely on digital technologies to manage their mental health and the importance of designing future digital interventions that are deeply rooted in adolescents’ lived experiences and real-world context. In general, interventions must account for the potential ambivalence toward behavior change, recognizing that motivation and engagement can vary widely, and prioritize privacy, accessibility, and emotionally responsive support. Our findings also point to areas in need of more research attention, particularly how AI systems respond to self-injury disclosures.

## Data Availability

The raw data supporting the conclusions of this article will be made available by the authors, without undue reservation.
