# Supplementary material for: Navigating self-injury in a digital world: adolescents’ perspectives on coping, help-seeking, and technology
Source: Front Digit Health. 2026 Aug 26;8:1905943. doi: 10.3389/fdgth.2026.1905943 (PMC13553821; doi:10.3389/fdgth.2026.1905943)
Supplement: Supplementary file 1 [file Datasheet1.pdf]

Developing a Digital Intervention for Adolescent Nonsuicidal Self-injury
Interview protocol (60 minutes)

Hi there, can you see and hear me, ok?

My name is [name] and I'm a researcher at Northwestern University

This session should take around 1 hour. Your participation is completely voluntary. This means you don't have to participate if you don't want to and if you do agree to participate, you have the right to only answer the questions you choose to. As mentioned in the consent form, I will be recording this session because recordings are helpful for me if I miss anything in my notes.

The potential risks of this research are minimal and confidentiality of private health information that you share with us will be maintained to the highest level and only accessible by me and my research team.

I do need to let you know that if you talk about current intentions to end your life or harm someone else, I may need to break confidentiality and disclose that information to keep everybody safe.

Any questions on that?

During the interview I'll ask you questions about the ways you manage your mental health, including your self-injury thoughts and behaviors, if and how you use technology as part of the process of managing self-injury thoughts and behaviors, and how you might imagine an app being helpful or harmful in this process. If at any point you feel uncomfortable answering questions please let me know and we can check-in, skip questions, or come back to them later.

Any questions?

Ok, I'll start the recording.

I'd like to start by asking you about your experience with self-injury.

- For some people, self-injury is understood to be a coping strategy. In fact, one of the most common reasons self-injuring is to cope with negative feelings or experiences – but this isn't the case for everyone.
- Do you see your NSSI as a way of coping with things?
  - If yes, how so?
  - If no, thanks for sharing that. Could you tell me a little more about your experience of self-injury then?
- Have you tried anything to manage your self-injury thoughts or urges?
  - If yes:
    - What helps?
    - What hurts or doesn't help?
  - If no:
    - Are there specific reasons why you haven't tried anything yet?
    - Do you wish to in the future?
- What are some of your concerns about self-injury currently? (e.g., these could be things like they cause tension in relationships, hurting yourself more than you intend, etc.)
- Do you have any current goals related to your self-injury thoughts and behaviors?
  - If so, what are those goals?
  - Is there anything you're worried might get in the way of you achieving these goals?

Now, I'm curious to learn more about your use of technologies…

- Do you use any technologies to support your mental health and well-being (this includes self-injury specifically)?
    - If yes:
        - Could you tell me a bit about what you use?
        - When do you use these technologies?
        - Does it help? Why or why not?
    - If no:
        - Have you ever used any technologies to support your mental health or well-being?
            - If yes:
                - Could you tell me a bit about what you've used?
                - When did you use these technologies?
                - Did it help? Why or why not?
                - What made you stop using [technology]?
            - If no:
                - Why not?

Next, I'm going to ask you about some specific uses of technology. Some people use technologies like apps, social media or online communities or forums to **talk about things they are going through** or to **seek support**.

- Have you ever used technologies to talk about, or seek support, for **mental health or self-injury**?
    - If yes:
        - What technologies have you used to talk about, or seek support for, your mental health or self-injury?
        - Was this for self-injury specifically?
        - Do you currently use these technologies?
        - What do [did] you do? When do [did] you use these?
        - Do [did] you find this helpful? Why or why not?
    - If no:
        - Why not?

Some people use technologies to **access information about mental health or self-injury, to learn more, and to seek out alternative coping strategies.**

- Have you ever used technology to get information about how to manage your mental health or self-injury?
    - If yes:
        - What technologies have you used to learn more?
        - What types of information did you seek?
        - Do you currently use these technologies?
        - Did you ever used that information to manage your mental health / self-injury?
        - Was it helpful/harmful?
    - If no:
        - Why not?

Are there any ways you'd imagine wanting to use technologies, such as an app, to support you in your self-injury self-management?

- What types of technologies would you want to use? [if participant identifies one technology use that language throughout]
- What would you want it to do?
- What types of content, or information, would you want this [technology] to have?

- How would you like to interact with this [technology] (e.g., daily, weekly, through messaging, etc.)? How often?
- Would you want to use the [technology] alone or would you want to interact with others?
    - Who would you want to interact with?
- Would you ever want to share information from the [technology] with others? (e.g., Friends, Parents/guardians, Other trusted adult, Doctors, Mental health professionals)
    - If yes:
        - How do you imagine sharing that information? What would the goal of sharing be? OR how do you imagine that sharing would help you?
    - If no:
        - What are your concerns with sharing information from the [technology] with others?
- How do you feel about AI? [Added after this came up organically]
